# The Choice of Operation for Prostatic Enlargement

**Published:** 1902-05-17

**Authors:** 


### THE CHOICE OF OPERATION FOR PROSTATIC ENLARGEMENT.

The increasing acceptance which has of late been accorded to the operation of prostatectomy renders the choice of operation, as between prostatectomy and prostatotomy, a problem of some importance. Dr. Ramon Guiteras,[1] of New York, says that the indications which determine a choice between prostatectomy and prostatotomy (Bottini's operation) are the age of the patient, the size of the gland, and the condition of the bladder and kidneys. As a general rule it may be said that cases in which there are slightly diseased kidneys, and prostates that do not feel large on rectal examination, although they cause considerable urethral impediment, are fitted for prostatotomy; while, on the other hand, middle-aged men with large prostates, as felt through the rectum, with good kidneys and sound bladders are subjects for prostatectomy. Age is an important consideration, for the older the patient the more liable he is to die from shock and asthenia, so that in a very old man, if the prostate be of the right variety, a Bottini operation should be performed. Old age, however, is not an absolute contradiction to prostatectomy. In regard to the influence which the shape and size of the prostate has upon the choice of operation, it may be said that cases in which the prostate feels very large by rectum are suitable for enucleation; while those in which the prostate does not feel considerably enlarged on rectal touch but which offers a distinct impediment to the introduction of instruments into the prostatic urethra, and in which there is consequently a considerable amount of residual urine, are best suited for prostatotomy. In this class of cases are those that present the so-called "prostatic bar" which is so often spoken of in connection with the subject of prostatic hypertrophy.

[1] New York Med. News, April 26.

